# Strategies of care for adolescent users of crack undergoing treatment

**DOI:** 10.17533/udea.iee.v37n3e12.

**Published:** 2019-10-23

**Authors:** Juliane Portella Ribeiro, Giovana Calcagno Gomes, Marina Soares Mota, Elitiele Ortiz Santos, Adriane Domingues Eslabão

**Affiliations:** 1 Nurse, Ph.D. Adjunt Professor, Universidade Federal de Pelotas; Brasil. Email: ju_ribeiro1985@hotmail.com Universidade Federal de Pelotas Universidade Federal de Pelotas Brazil ju_ribeiro1985@hotmail.com; 2 Nurse, Ph.D. Associated Professor, Universidade Federal do Rio Grande, Brasil. Email: giovanacalcagno@furg.br Universidade Federal do Rio Grande Universidade Federal do Rio Grande Brazil giovanacalcagno@furg.br; 3 Nurse, Ph.D. Adjunt Professor, Universidade Federal do Rio Grande, Brasil. Email: marinamota@furg.br Universidade Federal do Rio Grande Universidade Federal do Rio Grande Brazil marinamota@furg.br; 4 Nurse, Ph.D. Professor, Universidade Federal do Rio Grande do Sul, Brasil. Email: elitiele_ortiz@hotmail.com Universidade Federal do Rio Grande do Sul Universidade Federal do Rio Grande do Sul Brazil elitiele_ortiz@hotmail.com; 5 . Nurse, Ph.D candidate. Coordenadora da Atenção Básica do município de Capão do Leão, RS. Brasil. Email: adrianeeslabao@hotmail.com Prefeitura Municipal de Capão do Leão Brazil adrianeeslabao@hotmail.com

**Keywords:** Adolescent, Crack Cocaine, Street Drugs, Mental Health, adolescent health services, qualitative research., Adolescente, Cocaína Crack, Drogas Ilícitas, Salud Mental, Servicios de Salud del adolescente, investigación cualitativa.

## Abstract

**Objective.:**

To analyze the care strategies of the adolescent crack user under treatment.

**Methods.:**

Study of qualitative approach of descriptive type. The participants were 20 professionals from the *Centro de Atenção Psicossocial Álcool e Drogas* (Center for Psychosocial Care Alcohol and Drugs) and 10 professionals from the *Centro de Atenção Psicossocial Infanto-juvenil* (Center for Child Psychosocial Care) in a municipality in the interior of Rio Grande do Sul (Brazil). Data collection occurred through semi-structured interviews and the data were submitted to Thematic Analysis.

**Results.:**

It was identified the development of strategies in four scopes: attractive activities in specialized services, the building of bonds between the team and the adolescent, the inclusion of family in the care and the intersectoral work.

**Conclusion.:**

Care strategies, directed to and that address the needs of adolescents, contribute to better adherence to treatment and social reintegration.

## Resumen

**Objetivo.:**

Analizar las estrategias de cuidado del adolescente usuario de crack en tratamiento.

**Métodos.:**

Estudio con abordaje cualitativo de tipo descriptivo. Los participantes fueron 20 profesionales de un Centro de Atención Psicosocial Alcohol y Drogas y 10 profesionales de un Centro de Atención Psicosocial Infanto-Juvenil de un municipio del interior de Rio Grande do Sul (Brasil). La recolección de la información se realizó mediante entrevistas semiestructuradas y los datos se sometieron a análisis temático**.**

**Resultados.:**

Se identificó el desarrollo de estrategias en cuatro ámbitos: actividades atractivas en los servicios especializados, la construcción de vínculos entre el equipo de salud y el adolescente, la inclusión de la familia en el cuidado, y el trabajo intersectorial.

**Conclusión.:**

Las estrategias de cuidado, direccionadas a cubrir las necesidades de los adolescentes, contribuyeron a una mejor adhesión al tratamiento y a la reinserción social.

## Introduction

Adolescence is a fundamental stage in people's development, characterized by biological and psychosocial changes that affect all aspects of adolescent life. It is a vital phase that includes moments of choices, decisions, and experiences that often determine future development.(1) In this context, experiences of both licit and illicit drug use often arise, which may lead to abuse and dependence. One of these illicit drugs is crack, which began to circulate in Brazil approximately 30 years ago, covering an increasing number of adherents to its habit, including the adolescent public.(2) According to the *Pesquisa Nacional de Saúde do Escolar* (National Health Survey of the School Student) conducted by the *Instituto Brasileiro de Geografia e Estatística* (Brazilian Institute of Geography and Statistics) between 2012 and 2015, there was an increase in the percentage of 9th grade students, which equates to the starting year of highschool, who tried illicit drugs (marijuana, cocaine, crack, *cola*, *loló*, *lança-perfume*, ecstasy, among others), going from 7.3% to 9.0%. Of these, 5.5% used crack in the last 30 days prior to the survey.(3)

For this reason, crack consumption and its implications in adolescence has been a matter of concern in society due to clinical and social problems that happen in a precocious manner. In adolescent health, crack use can cause psychotic disorders, cognitive impairment, mood and behavioral changes, and cardiorespiratory problems.(4,5) In addition, adolescent crack users are exposed to situations of violence, criminality, and also dropping out from school, leaving them socially vulnerable as well.(5,6) Thus, the complexity of the consequences related to the abusive use of crack in adolescence and the social vulnerability to which many adolescents and young people are exposed, demands new ways of producing health, with priority for integrated actions within the scope of intersectoral public policies and networking aimed at efforts for prevention, treatment adherence and harm reduction.

The current global health strategy, which covers the period 2016-2030, includes adolescents as a priority target for actions, proposing an agenda aimed at guaranteeing health rights, for physical and mental well-being, and the promotion of healthy behavior, preparing them to develop their full potential in adulthood.(1) In Brazil, governmental actions in the area of ​​drugs emphasize the construction of the *Rede de Atenção Psicossocial* (RAPS, Psychosocial Care Network) for people in psychological distress and with needs arising from the use of crack, alcohol and other drugs. The RAPS strengthens the conception of continuous care with guaranteed access and integration of the points of care, especially for children, adolescents and young people due to their vulnerable condition.(7) At RAPS, the referral services for specialized care for adolescent crack users are *Centros de Atenção Psicossocial* (Psychosocial Care Centers), in their child or adult mode, aimed at users of alcohol and other drugs. Adolescents in these services should be targeted for prevention, harm reduction, intersectoral actions, and social reintegration.(7)

Although treatment guidelines are established in the public sphere and the extent of the theme has grown, mental health services face difficulties in presenting resolute proposals to the adolescent user of crack, alcohol and other drugs. It is verifiable that beyond being incipient the installation of specialized services for this population, both the *Centro de Atenção Psicossocial Infanto-juvenil* (CAPSi, Child Psychosocial Care Center) and the *Centro de Atenção Psicossocial Álcool e Drogas* (CAPSad, Psychosocial Care Center for Alcohol and Drugs), there is a difficulty in the organizing of and planning of specific actions the demands of the adolescent substance user public.(8-10) Thus, by not having their specificities considered, the adolescent has poor adherence to the actions of the service, difficulty in linking teams and frequent relapses, constituting an important barrier to treatment.(9-11) This scenario highlights the need to understand the care strategies for adolescent crack users, favoring the organization of teams to offer actions that can assist in treatment adherence and social reintegration. Therefore, the article aims to analyze the care strategies of adolescent crack users undergoing treatment.

## Methods

This is a descriptive study with a qualitative approach to data, linked to a broad research project entitled ‘*(Des)caminhos percorridos pelo adolescente usuário de crack na rede de atenção psicossocial: contribuição para a Enfermagem*’ (or ‘(Mis)paths coursed by the adolescent crack user in the psychosocial care network: contribution to Nursing), developed at the *Centro de Atenção Psicossocial Álcool e Drogas* (CAPSad, Psychosocial Care Center for Alcohol and Drugs) and the *Centro de Atenção Psicossocial Infanto-juvenil* (CAPSi, Child Psychosocial Care Center) of a medium-sized municipality in the interior of Rio Grande do Sul (Brazil) that is part of the Crack Program, it is possible to win. The project was submitted to the *Comitê de Ética* (Research Ethics Committee) and approved following the *Certificado de Apresentação para Apreciação Ética* (Certificate of Presentation for Ethics Appreciation). In order to ensure ethical principles for research involving human subjects, participants were included in the study only after being advised about the study's objectives and methodology and expressing their agreement to participate by signing the Free and Informed Consent Form. In addition, the anonymity of the participants was preserved by using the letter P succeeded from the interview number and service to which they belong.

Twenty CAPSad professionals and 10 CAPSi professionals participated in the study. The selection of participants was intentional, according to the inclusion criteria and research objectives. Namely, the inclusion criteria were: being a mid-level or higher worker who is part of the CAPSad or CAPSi multiprofessional team; have at least six months of experience in the service. Professionals on vacation or sick leave during the data collection period were excluded. The number of participants was defined by the data saturation defined when, in the researcher's evaluation, a certain redundancy or repetition occurs, and it is not considered relevant to persist in the data collection. Data collection took place in the first half of 2017, through semi-structured interviews conducted by a single interviewer, questioning them about the care strategies offered to the adolescent crack user under treatment. In view to participants' privacy, the interviews were conducted in rooms available at the services, respecting the functioning of CAPSad and CAPSi. In order to preserve the original content and increase the accuracy of the data obtained, the interviews were captured by an audio recorder and later transcribed in full. For the organization and processing of the data, the Nvivo 11 software was used, and subsequently analyzed and categorized according to the Thematic Analysis. From the organization and analysis of data emerged the following categories: Attractive Therapeutic Activities; Bond building; Inclusion of the family; and Intersectoral Work.

## Results

From the data analysis, it was identified the development of strategies in four areas: attractive activities in specialized services, the building of bonds between the team and the adolescent, the inclusion of the family in the care and intersectoral work.

### Attractive Therapeutic Activities

The adolescent public is considered more agitated, with more energy, showing greater adherence to activities performed outside the service, in community resources such as squares, libraries and events. In CAPS, stands out the therapeutic workshops of music and gym. In this sense, one identifies as a strategy the attractive therapeutic activities, directed to the needs of this audience: *Upon arrival, we make a nursing consultation, always asking: what does he like to do, because CAPS activities are not very targeted at teenagers, for example, they are not about painting, nor about making crafts. They still do, but that's unattractive. So we try to fit them into the music classes, the gym, which they like, to spend a lot of energy. [...] There are teenagers who need the street, so there have to be street activities. We use a lot of street resources, because there is the square, the library. Whatever is an event that we can participate we take them. This is not easy, activities need a change all the time* (P7 CAPS AD)*. The teenager comes with a whirlwind of things along, so the team needs to have the profile to work with them. They have much more energy, much more agitation, need to have much more activities, need to have specific activities that compensate, have verbal handling that is 24 hours* (P18 CAPS AD)*. [...] or because of abstinence, tolerance, they end up not having as much focus on the proposed activities so they have to have a bigger unfolding, more to offer, more variety of activities because really the focus is not so long and the whole question the impulse to leave the place, to be stuck* (P4 CAPS i)*.*

Thus, it is necessary to broaden the menu of activities offering modalities such as: internet access, video games, sports in multi-sport courts, socialization activities in the community and pedagogical accompaniment: *To improve care would be necessary other modalities and activities such as access to internet and video games, to keep the teenager in service. In addition, spaces would be indispensable for sports, such as sports courts* (P9 CAPS AD)*. We're dealing with teenagers and they have a lot of energy, not only energy for being a teenager, but this whole lust that comes with substance use, so keep them indoors, just television, just play, just handle verbal is not enough, they need sports, they need pedagogical accompaniment. They can't stay closed, the world is out there waiting for them. This treatment can not be totally closed here between four walls, this treatment will be part of a walk in a square, to take in a historic center, rescue what he lost* (P17 CAPS AD)*.*

### Bond building

Care for adolescent crack users requires the building of bonds, so that the team develops strategies to establish trust, management and patience, respecting the adolescent's moments: *In care they are very suspicious, so you need to gain their trust* (P14 CAPS AD)*. You have to have management, you have to have patience, you can't just come and go, you have to have a bond [...]. It's a building job, bond establishing. He has to realize that he has a bond with this team and that he can trust that team because only then can we get him to go to the host unit* (P16 CAPS AD)*.* The bond with the team facilitates the identification of social needs and adherence to treatment, and the turnover of professionals can interfere in this process*: [...] here we work with the user, we work with bond and, unfortunately, they come and go. So today he comes and talks to me, tomorrow is you. This is very bad, this whole turnover. Teenagers have ties with us and soon we change, they have a lot of losses and that is also very bad* (P7 CAPS AD)*. [...] We discover things like this little by little, as you go, they get confidence* (P9 CAPS i)*.*

### Family Inclusion

The care for the adolescent crack user involves the inclusion of the family member, since the adolescent is underage, and especially because the focus of the problem is often found in the family. The participation of the family member is seen as one of the necessary aspects for the progress in treatment and the family group as an important therapeutic device of care: *We try to locate family members and always involve the family member because they are underage* (P19 CAPS AD)*. In UAI there are two days of visit, and what we see, that the family puts them inside the UAI [...]. So if we don't do this strong, well-done work with this family, we can't see progress* (P7 CAPS AD)*. [...] the whole family issue has to be worked out [...]. This is very related to the lack of family structure with drug use, so I think the conduct of the team has to be differentiated* (P6 CAPS i)*. Also interesting is the teen-only family group that is run by a social worker. There is that family group there because it is no use treating only the teenager, often the problem is there in the family* (P2 CAPS i)*.*

### Intersectoral work

Adolescent crack users are in vulnerable environments, exposed to conflicting family relationships and street life. Thus, it is necessary to work in intersectoral network through dialogue and joint actions between health, social assistance, tutelage council, the judiciary sector and partnerships with community devices aiming at offering possibilities of social reintegration: *I think it has to be much further, because Only the CAPS and the Childcare Unit will never meet all the demand, it has to be an intersectoral work. These teenagers, mostly, have broken families, sometimes have no father, no mother, or only mother, sometimes parents are users, sometimes traffickers, sometimes they're living on the street, so they demand from other sectors* (P8 CAPS AD)*. I think one thing that we have been focusing a lot and that is very difficult is the intersectoral partnerships, so to get courses, workshops, gymnastics, pilates, box, computer course, training course, internships, that kind of thing, you should prepare them better and that you could really offer them new prospects for reintegration* (P18 CAPS AD)*. I think it is the services that sit and talk, the RAPS points of attention that all work with children and youth [...], and thus it has to be intersectoral including, not only RAPS. Intersectoral, because we will work together with the council, we will work together with the judiciary, we will work together with CRAS, many times we will work with a teenager who is using marijuana, for example, but the mother and the father so using heavier drugs more periodically then, you have to work with the network if you don't work with the network and then so when I say work with the non-model network alone. Then the damage reduction or CAPSad be the articulator, but they have support from other services as well [...]* (P5 CAPS i)*.*

## Discussion

Adolescents and young people constitute a population group that requires new modes of health care. The health problems evidenced in this phase are largely due to habits and behaviors that, in certain situations, make them vulnerable. In this context, attention to adolescents should be guided by comprehensiveness, impressing a look at the different aspects of adolescent life and their specificities, which requires reorganization and planning of actions directed to this audience.(12) In this study, care strategies for adolescent crack users within the scope of RAPS include the development of dynamic therapeutic activities involving music, sports, the use of the internet and video games, and especially insertion activities in community spaces. These activities are considered more attractive for maintenance treatment.

Games and music are also identified in another study as potent therapeutic resources in the treatment of adolescent psychoactive substance users.(11) These strategies make it possible to work in a playful and attractive way on aspects related to cognition, social interaction, expression of feelings, simulations of real life situations and exposure to situations of vulnerability. In addition, the study underscores the importance of specific actions and strategies that consider the socioeconomic and cultural context, aiming to assist adolescents in establishing and empowering the protagonism of their lives. Practices involving playfulness, fantasy, communication and imagination are also means to clarify doubts and provide guidance on the relationship of pleasure and suffering caused by drug use. It is identified that these activities are most effective when built in groups, because young people, by making analogy of biographical situations and sharing experiences, can better work with their own issues.(13) The organization of the service in a way that is more attractive to adolescents goes through a construction process, and it is first necessary to be closer to the reality of young people in order to comprehend in a broader way the reality in which they are inserted, and thus develop strategies that can develop critical thinking and reflection on the problems experienced.(10)

It is identified in the present study that the organization of the service to offer such activities is still a challenge, and CAPS needs to advance in this aspect. The activities traditionally performed in specialized mental health services do not meet the needs of this public. These data corroborate research that points to a deficit in relation to the organization of *Centros de Atenção Psicossocial* (Psychosocial Care Centers) to attract adolescents, which may reflect on the follow-up of therapy and treatment losses.(8,9) It is noteworthy that the *Política Nacional de Saúde Mental* (National Mental Health Policy) signals the structuring and strengthening of specialized services in the mental health network aiming at an organization and qualification of the actions provided. However, in addition to the more attractive environment for this audience, it is necessary to consider the singularities of each adolescent, prioritizing a different attention in the construction of each therapeutic plan. Thus, therapeutic activities that are attractive to adolescents should be dynamic and targeted, which arouses their interest and motivation. We highlight activities such as music, sports, use of new technologies, and above all, activities performed outside CAPS, in the community, in spaces of socialization, leisure and culture. In this sense, it is important to know the needs and community resources, integrating actions in the territory of life of adolescents.

The bond construction as another care strategy requires management, patience and establishment of trusting relationships to adhere to the proposed strategies and social reintegration. Researchers point out that bonding between the adolescent and the team is one of the main factors in adherence to treatment, favoring commitment to therapeutic activities and changes in behavior regarding drug use.(14) In caring for adolescent crack users, bonding develops respect for choices, encourages participation in treatment, and moments of listening without prejudice and judgment so that adolescents perceive, in the service, a place of inclusion and acceptance of their needs beyond drug use. Many adolescent drug users face taboos and prejudices until they come to health services for help, so it is important for staff to adopt a cordial, understanding attitude to build trust. This mode of attention aims to break with the traditional logic of attention, which is still very much present in practice, where it is observed the imposition of norms and behaviors that place the adolescent in a position of inferiority and passivity, removing not only freedom of choice but also the responsibility for their actions.(15)

However, it is worth noting that the construction of the bond as a therapeutic resource is a complex process that requires, in addition to technical skills, the organization of the service provided to it, as well as the reduction of the turnover of professionals in mental health services, a factor that already highlighted in previous research.(16) Inclusion of the family in treatment was also identified as a care strategy for adolescent crack users within the scope of RAPS. The present study demonstrates that the progress in therapy is related to the inclusion of the family member in the treatment, since it is within the family structure that many problems are found, which may be related to drug abuse and, therefore, need understanding. and approach in this area.

Adolescent drug use may be related to the exposure of children and adolescents to many problems that permeate a disrupted family environment. In this regard, the research highlights the lack of coexistence or lack of dialogue with parents or guardians, the use of drugs in the family, facilitated access to drugs in this environment and experiences of aggression in the family environment.(17,18) In this sense, the use of crack in adolescence is not an isolated problem, but related to different factors, in which the family context has an important influence. This aspect alerts to the situation of family members and highlights the reflection of weaknesses in family dynamics in the life and illness of their members.(19) It is not uncommon for RAPS professionals to serve members of the same family in different mental health services and witness the perpetuation of the consequences of a problematic family context across different generations.

Families' difficulties in dealing with the problems of adolescent drug abusers are mainly related to a scarce support network in the community that gives them proper support. This fragility makes it difficult for the family members to be co-responsible for adolescent care and generates a feeling of powerlessness and helplessness in the family, which often causes the family member to seek institutionalization in shelters or psychiatric hospitals, an option that has not been effective.(8) Given this situation, it is not up to professionals to judge or blame families, but rather to include them as a target of care and partners in the treatment of adolescent crack users. Family support is fundamental for physical and social restoration, as well as for continued treatment.(20,21) On the other hand, the family integration deficit, with discussions, discrimination and fights, stimulates the use of alcohol and other drugs, distorting and destroying the self-confidence and self-esteem of the members.(21) The inclusion of the family member in the treatment becomes relevant due to the weaknesses identified in this context that require understanding and assistance. Thus, we suggest strategies that aim to strengthen the role of the family and family relationships such as family group activities and individual family care in specialized services. Therefore, it is the responsibility of the services and public policies to promote family care and conditions for their participation and partnership in care, even if the difficulties may be great. It is necessary to know the family's experiences, feelings, and parents' experience regarding the use of crack and other drugs. This strategy helps to overcome anxieties and doubts so that they are encouraged and empowered to act in the process of social reintegration of their children.(18)

Intersectoral work was also identified as one of the demands of adolescent crack users, constituting an important strategy for comprehensive care and action on social vulnerabilities in which many adolescents are inserted. Sectors such as health, social assistance, guardianship council, the judiciary and community devices should compose this partnership, through dialogue and joint actions, aiming at offering possibilities of social reintegration. This finding is in line with other studies that highlight the intersectoral articulation as one of the possibilities to broaden the action on the needs of adolescent crack users and overcome the difficulties faced in caring for this public.(18,19)

The political and institutional field stresses the need for an intersectoral and multidisciplinary approach in the care of adolescent crack users, with development not only of clinical actions, but also related to family, community, school, housing, culture, drug trafficking and violence, emphasizing the harm reduction strategy as the axis of actions in the SUS. Despite the recognition of the necessary articulation in an intersectoral network, what is currently observed are sectoral or institutional actions that contribute little to the management of the phenomenon of drug use in adolescence. In general, it is clear that the health, social assistance, school and judiciary sectors interfere at different levels and situations, with a delegation of responsibilities that sometimes falls on one sector, sometimes on the other.(5,23)

The results of this sectorial work are visible as new data point to the increase in crack consumption in adolescence and to the difficulties in the treatment and rehabilitation of users and their families who deal daily with the problem.(18,22) According to the participants of this study, intersectoral networking involves spaces for sharing, dialogue between the different actors that are part of the care of children and youth, having as articulators of the network CAPSad and Harm Reduction. In this conception, partnerships with social devices in the territory are also essential, with the offer of courses, training, therapeutic workshops, internships, sports, as a way to offer different perspectives of social reintegration through an intersectoral practice.

These data corroborate the research that highlights the partnerships in the territory as essential for network design, giving an intersectoral practice on health needs. However, the study points out that these resources are little explored, requiring greater involvement of professionals, including partnerships, funds and interests in the implementation and maintenance of these spaces.(24) In this sense, it is understood that the intersectoral network in the care of adolescent crack users is a task under construction, requiring the involvement of professionals, management support, material resources and qualified human resources in order to expand the possibilities of its implementation, producing more significant results in the social reintegration of adolescent crack users. From the above, it is considered that the health network, through strategies directed to adolescents can help them in the process of treatment and social reintegration, preventing them social and health damage in their current phase and in adulthood.

The results of the present study allow us to conclude that the care provided to adolescent crack users under treatment involves the development of strategies in four areas: attractive activities in specialized services, the building of bonds between the team and the adolescent, the inclusion of the family in care, and intersectoral work. These care strategies directed to the needs of adolescents contribute to a better adherence to treatment in the Psychosocial Care Centers and to their social reintegration.

In the present study we sought to deepen the most outstanding demands at the present time by workers of specialized services. However, it is understood that there are different demands for care when considering this theme regarding adolescents and drug use, such as the prevention demands that may be the target of future studies.

As a limitation of the study, it is pointed out that it was performed in two services that make up the RAPS, not covering other emblematic services in the care of adolescents who use crack, alcohol and other drugs. For this reason, it is recommended that new research include the analysis of other services and sectors such as guardianship council, education and justice that are directly involved in the care of young people using crack, contributing to the knowledge of new practices that address the complexity of care of this audience.
